# The Global Epidemiology of Syphilis in the Past Century – A Systematic Review Based on Antenatal Syphilis Prevalence

**DOI:** 10.1371/journal.pntd.0004711

**Published:** 2016-05-11

**Authors:** Chris Richard Kenyon, Kara Osbak, Achilleas Tsoumanis

**Affiliations:** 1 HIV/STI Unit, Institute of Tropical Medicine, Antwerp, Belgium; 2 Division of Infectious Diseases and HIV Medicine, University of Cape Town, Cape Town, South Africa; 3 Clinical Trials Unit, Institute of Tropical Medicine, Antwerp, Belgium; University of Tennessee, UNITED STATES

## Abstract

**Background:**

How can we explain the uneven decline of syphilis around the world following the introduction of penicillin? In this paper we use antenatal syphilis prevalence (ASP) to investigate how syphilis prevalence varied worldwide in the past century, and what risk factors correlate with this variance.

**Methods:**

1) A systematic review using PubMed and Google Scholar was conducted to identify countries with published data relating to ASP estimates from before 1952 until the present. Eleven countries were identified (Canada, Denmark, Finland, India, Japan, Norway, Singapore, South Africa, United States of America (USA), United Kingdom (UK) and Zimbabwe). The ASP epidemic curve for each population was depicted graphically. In South Africa and the USA, results are reported separately for the black and white populations. 2) National antenatal syphilis prevalence estimates for 1990 to 1999 and 2008 were taken from an Institute for Health Metrics and Evaluation database on the prevalence of syphilis in low risk populations compiled for the Global Burden of Diseases study and from a recent review paper respectively. National ASPs were depicted graphically and regional median ASPs were calculated for both time periods. 3) Linear regression was used to test for an association between ASP in 1990–1999 and 2008 and four risk factors (efficacy of syphilis screening/treatment, health expenditure, GDP per capita and circumcision prevalence). WHO world regions were included as potential explanatory variables.

**Results:**

In most populations, ASP dropped to under 1% before 1960. In Zimbabwe and black South Africans, ASP was high in the pre-penicillin period, dropped in the post-penicillin period, but then plateaued at around 6% until the end of the 20th century when ASP dropped to just above 1%. In black Americans, ASP declined in the post penicillin period, but plateaued at 3–5% thereafter. ASP was statistically significantly higher in sub-Saharan Africa in 1990–1999 and 2008 than in the other world regions (*P* < 0.001). On multivariate analysis in both time periods, ASP was only associated with residence in sub-Saharan Africa.

**Conclusions:**

Further research is necessary to elucidate the reasons for the higher prevalence of syphilis in sub-Saharan Africa.

## Author Summary

Syphilis rates have varied tremendously between different populations around the world. We conducted a systematic review of syphilis prevalence in pregnant women in 13 populations with available data for the last 100 years. Our findings were that in most populations syphilis prevalence dropped to under 1% before 1960. In the 2 populations from sub Saharan Africa, the syphilis prevalence remained around 6% until 50 years after the introduction of penicillin. Other systematic reviews were utilized to provide syphilis prevalence estimates for all countries with available data for the periods 1990–1999 and 2008. We assessed if there was a correlation between national syphilis prevalence in these periods and five explanatory factors. Only residence in sub-Saharan Africa was associated with syphilis prevalence in both time periods. These findings, considered in conjunction with other types of evidence we review, such as the strong correlations at population level between syphilis prevalence and those of Herpes Simplex Virus-2 prevalence and HIV prevalence, suggest that common risk factors may underpin the spread of all three of these sexually transmitted diseases. Establishing what these factors are is of great importance to improve the health of highly affected populations such as those in sub-Saharan Africa.

## Introduction

Can a meaningful pattern be discerned in the large variations in syphilis rates over the last century? A first step toward answering this question is the mapping of syphilis rates across time and place followed by correlation analyses with possible explanatory variables [1]. Numerous papers have published descriptions of longitudinal changes in reported cases of syphilis within countries that are associated with events such as the introduction of penicillin, social disruption associated with wars or the collapse of the Soviet Union [2,3]. Other analyses have investigated reasons for the higher incidence in subpopulations such as men who have sex with men (MSM) [4] and non-Hispanic blacks in the United States of America (USA) [5]. The World Health Organization (WHO) and others have published estimates of world regional syphilis prevalence for 1998, 2001, 2005 and 2012 [6–9] and national estimates of antenatal syphilis prevalence for 2008 [10].

This paper extends this descriptive epidemiology of syphilis in three ways. Firstly, we conduct a systematic review of countries with published antenatal syphilis prevalence (ASP) estimates from pre-1952 till the present. We find 13 populations in 11 countries with available data over the last century and use this to chart variations in these populations’ ASP trajectories. Secondly, we generate ASP prevalence estimates by country for the time period 1990–1999. The prevalence of syphilis plummeted in a number of hyper endemic countries around the time of the AIDS epidemic in the late 1990s. This decline was in part due to the widespread introduction of the syndromic approach to STI management [11] and in part due to the effect of AIDS mortality breaking up sexual networks [12–15]. As a result, correlation analyses of risk factors associated with national ASP from 1990–1999 would be less likely to be affected by this effect of AIDS mortality than those from 2008. Thirdly, we use the 1990–99 and 2008 national ASP estimates to assess if four risk factors, efficacy of syphilis screening/treatment, health expenditure, GDP per capita and circumcision prevalence, are correlated with ASP in these two time periods.

Accurate mapping requires a measure of syphilis that can be meaningfully used to compare syphilis rates between populations. The number of cases of early stage syphilis per year reported as part of national surveillance programs (case-reporting-based syphilis incidence—CSI) is a commonly used variable and has been shown to be useful to compare syphilis trends over time within countries [3]. Case reporting is however beset by numerous problems: many cases of syphilis are asymptomatic [6,16], health seeking behavior varies between locales [17] and the completeness of reporting varies tremendously between different types of institutions [18], regions within countries [18] and between countries. A twenty four country study from Europe found that syphilis case surveillance coverage varied from below 10% to over 75% in these developed countries [19]. In addition, there are considerable differences in how sexually transmitted illnesses (STIs) are classified (such as syndromically or etiologically) [6] and in the accuracy of diagnosis of syphilis when the etiological system is used [15,20].

As a result, the CSI indicator is suboptimal for comparing syphilis rates between populations [10,17,21,22]. The WHO has suggested that it should continue to be used to provide minimum estimates of incidence in low prevalence countries [6]. In 1960, Guthe noted the deficiencies of CSI and proposed that “the most useful index of the prevalence of syphilis in a population is—for obvious reasons—the sero-reactor rate in pregnant women [21].” The WHO has recommended the use of ASP as an indicator of syphilis prevalence in high prevalence countries [6]. In this paper we take up this suggestion to use antenatal syphilis prevalence (ASP)—the serologically determined prevalence of syphilis in antenatal populations—to further describe the global epidemiology of syphilis.

## Methods

### Systematic review: longitudinal changes in ASP in 13 populations

#### Search strategy and selection criteria

**Fig 1:**
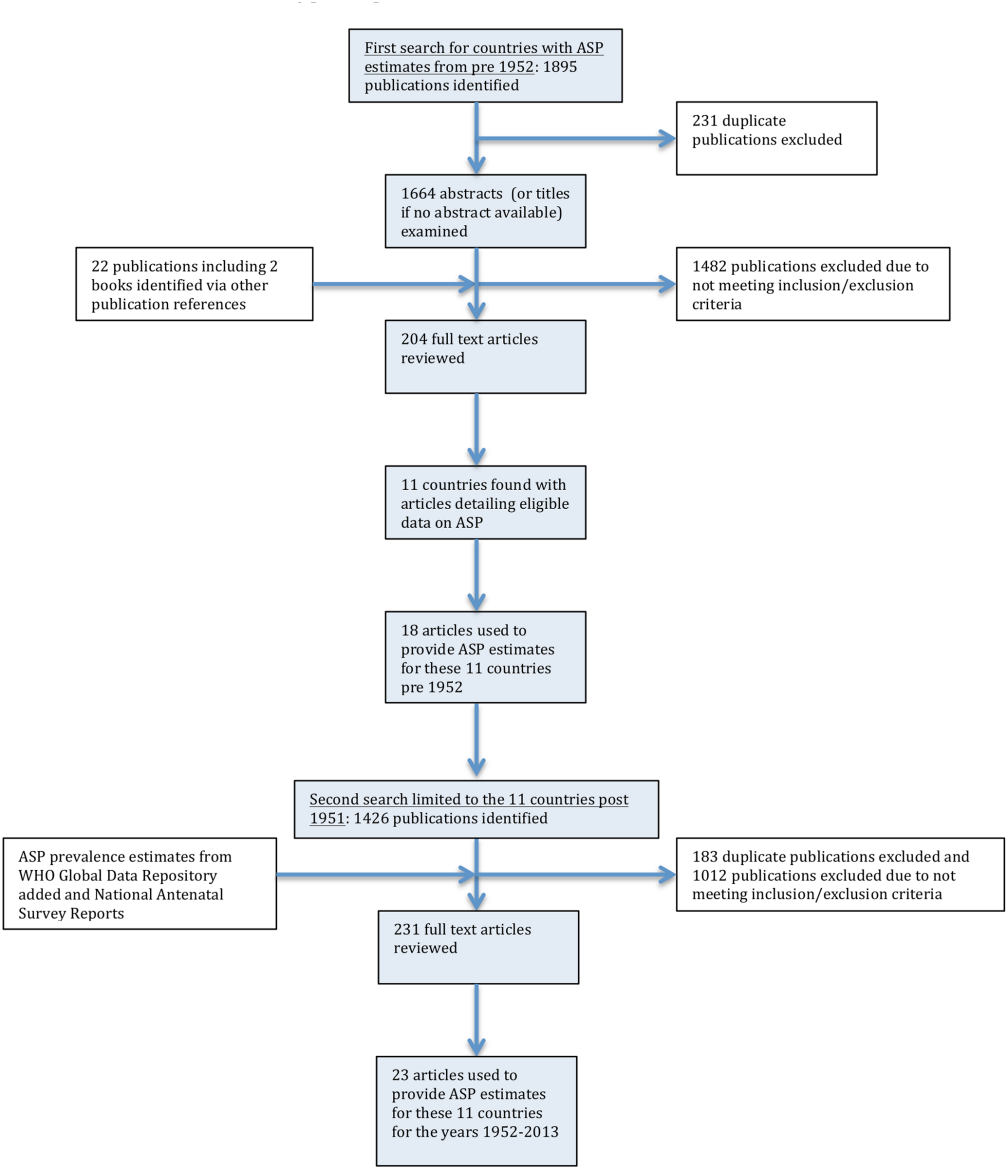
Flow chart showing selection of publications from the literature search for antenatal syphilis prevalence estimates.

The search was conducted in two stages (Fig 1):

i) In the first stage we sought to find as many countries as possible with published ASP estimates from pre- 1952. We conducted a literature review using PUBMED and Google Scholar to find countries with sufficient ASP data from any time from 1951 or before until the present. The 1951 cutoff was chosen as a compromise between being relatively early in the penicillin period and late enough to include a sufficient sample size of populations. MeSH terms used in the PubMed search (search date: 10/09/2015) were ‘‘prevalence”, OR ‘‘incidence”, OR ‘‘antenatal” OR ‘‘pregnancy”, OR ‘‘pregnant”; AND: ‘‘syphilis”, OR ‘‘treponema pallidum”; filters: publication date from 01/01/1915 until 30/12/1960. Similar search terms were used for the Google Scholar search (search date: 15/09/2015).

#### Inclusion and exclusion criteria

Studies were eligible for inclusion if they reported the percentage of all pregnant women attending general antenatal services testing positive for syphilis and they specified the type of diagnostic test used. Studies only reporting on high-risk pregnancies were excluded. In addition, studies needed to give an indication of the geographical location for which the population was drawn. Estimated values of ASP were read from figures if exact numbers were not available. Only articles in English were considered eligible.

1895 articles were identified and 204 articles reviewed, 11 countries were identified as providing eligible ASP from pre-1952. Three countries were from Asia (India, Japan, Singapore), two from Africa (South Africa- SA, Zimbabwe), two from North America (Canada, United States of America- USA) and four from Europe (Denmark, Finland, Norway, the United Kingdom—UK).

ii) In the second stage the search was directed at finding all ASP estimates for these countries between 1952 and 2015. We used the same search terms as above but with the new dates as limits and the searches limited to these 11 countries. The searches were conducted on PubMed (search date: 29/09/2015) and Google scholar (search date: 30/09/2015). This search strategy provided us with 23 articles with ASP estimates for these countries post- 1951.

In the case of the USA and SA the results are reported separately by race. This is justified by the large variations in syphilis prevalence between races in these countries and the strong tendency to racially homophilous sexual partnering found in these countries [23,24]. In each case the comparisons are limited to blacks and whites. Other groups are excluded for a number of reasons including small sample sizes and in the case of Hispanics in the USA, the different ways the ‘Hispanics’ category is defined in different surveys [25–27].

In both stages of the search, prevalence that was weighted or adjusted to account for selection bias was used where reported. Whenever possible we used ASP that was determined via both a treponemal and nontreponemal test. If this was not possible we report the ASP ascertained via the treponemal or nontreponemal and specify the testing type used (Table 1). In addition to the ASP estimates we included three prevalence estimates of syphilis prevalence for women by race for the USA from three waves of the National Health and Nutrition Examination Surveys. These surveys are designed to provide nationally representative samples.

**Table 1:** Sources of Antenatal Syphilis Prevalence data used in eleven-country comparison.

Country	Sampling period	Study design, testing modality and study reference
Canada	1938	1000 successive antenatal women attending the Toronto General Hospital screened with WR [84]
	2008	Global Estimates of Syphilis in Pregnancy and Associated Adverse Outcomes Study [10]
Denmark	1936–38	All 2000 antenatal clients seen at the Rigs General Hospital, Copenhagen over a three year period were screened with the WR [85]
	1950–65	In 1950, 1955, 1960 and 1965 all antenatal women were screened for syphilis with a WR or equivalent test. Approximately 90 000 women were screened at each survey [3]
	2010	WHO Global Health Observatory Data Repository
Finland	1926–34	2 390 antenatal clients attending Maternal Health Centre, Helsinki, were screened for syphilis with a WR or Khan Test or both. Proportion of all antenatal clients screened not stated [86]
	1935–45	18 090 antenatal clients attending Maternal Health Centre, Helsinki, were screened for syphilis with a WR or Khan Test or both. Proportion of all antenatal clients screened not stated [87]
	1986	110 000 consecutive pregnancies over a 20 month period were tested with the Khan test [88]
	2008	Global Estimates of Syphilis in Pregnancy and Associated Adverse Outcomes Study [10]
India	1950,1955, 1956	All antenatal women attending the J.J Group of Hospitals in Mumbai in 1950 (n = 484), 1955 (n = 4020) and 1956 (n = 4162) were screened for syphilis with the Khan or VDRL test [89]
	1952	13 609 antenatal women attending antenatal clinics in 1952 in Madras tested via the WR [90]
	1953	11 736 unselected antenatal women attending clinics in Madras during 1953 were tested with the WR [90]
	1973	In 1973, 5 894 antenatal women were tested for syphilis with the VDRL and Khan tests. Syphilis was defined as testing positive with both tests [91]
	1980–84	7 992 randomly selected women attending antenatal clinics in Delhi between 1980 and 1984 were tested for syphilis with the VDRL [92]
	1996–2005	40 511 sera from women attending the antenatal clinic of Nehru Hospital, Chandigarh, 1996–2005 were tested for syphilis with a VDRL. Confirmation was via TPPA. 3 800 to 4 300 were tested per year and results reported by year [93]
	2009–12	WHO Global Health Observatory Data Repository
Japan	1951–56	Neither screening methodology nor sample size described. Only results of “mass-screening’ antenatal populations provided [94]
	2008	Global Estimates of Syphilis in Pregnancy and Associated Adverse Outcomes Study [10]
Norway	1941	A representative sample of 922 women from Southern Norway were screened for syphilis with the WR [95]
	1944–48	All midwifes and doctors attending antenatal women in the town of Bergen were asked to send blood specimens for syphilis screening on all antenatal women. 4961 of 10647 pregnancies had specimens submitted. These were screened with the WR and Kahn's standard reaction. Meinicke's clarification test was performed if necessary [96]
	1957	Between 1957 and 1958, 27 445 antenatal clients from Southern and Western Norway were screened for syphilis with Meinicke Clarification Test II, Bordet-Wassermann Complement-Fixation Test and the Wadsworth and Brown's Flocculation Test [97]
	1964 & 1978	Prevalence estimates from approximately 50 000 antenatal clients screened annually in the whole of Norway in third trimester with the VDRL/WR/Meinicke Flocculation Test. Positive results confirmed with TPI [98]
	2008	Global Estimates of Syphilis in Pregnancy and Associated Adverse Outcomes Study [10]
Singapore	1951–58	Antenatal cases seen at suburban and rural maternity clinics were tested for syphilis with the WR. Results are reported by year for 1951–58. Between 1 363 and 11 448 women were tested per year [99]
	1974	Antenatal women tested for syphilis with RPR and confirmed with TPHA. Sample size not stated [100]
South Africa	1938–1946	Kark reviewed South African syphilis prevalence in different populations for the period 1921–1946. 8 studies were performed exclusively in black antenatal populations between the years 1938 and 1946. All used a WR to estimate syphilis prevalence. We used the median ASP from these studies for the 1942 black ASP (Median 29.7, IQR 19.5–38, Range 11.4–40.5) [73]
	1949–1971	254 137 antenatal black women screened for syphilis with the WR at 6 clinics in Soweto, between 1949 and 1971. Results are reported per year. 3 795 to 15 583 women were tested per year [101]
	1991 & 1998–2011	In 1991 and from 1998–2011, South Africa included testing for syphilis in its annual antenatal HIV surveys. The methodology of these surveys has been described in detail elsewhere [102–107]. In summary, public sector antenatal clinics were sampled on a probability proportional-to-size basis and women attending these clinics for the first visit of their pregnancy were tested for syphilis using a RPR test. No confirmatory testing with treponemal-specific assays was conducted. Only in the 1991 survey were results presented broken down by race
	1972	2 495 unselected antenatal women (2 056 coloureds, 232 blacks, 201 whites) from Cape Town were tested with WR or RPR and confirmed with FTA. [108]
United Kingdom	1922	Wasserman reaction test was performed on 1 881 consecutive admissions to a maternity hospital in Glasgow in 1922 [109]
	1944–49	71 645 antenatal samples tested via the Wasserman reaction test [110]
	1953–59	Syphilis prevalence in antenatal women taken from 6 regions in England and Wales provided by the Department of Health. Figures are provided for each year and involved the testing of between 28 263 and 56 962 women per year [111]
	1959–68	42 404 antenatal women seen at Queen Charlotte’s Hospital, London, between 1959 and 1968 tested with VDRL. All positives confirmed with TPI or FTA [112]
	1969–71	64 404 antenatal women tested for syphilis in Glasgow laboratories between 1969 and 1971 with WR or VDRL and confirmed by TPI or FTA [113]
	1983–87	76 519 antenatal women screened for syphilis in the Oxford region between 1983 and 1987. Tested with TPHA [114]
	1996–2010	WHO Global Health Observatory Data Repository
USA	1937–38	The charts of all pregnant women between 1937–38 in the District of Baltimore had their charts reviewed to see if they had a serological test for syphilis (1335 whites and 668 blacks). In 46.7% of whites and 7.6% of blacks no test was performed [115]. ASP is defined as the percent of those with a positive serological test (WR or equivalent) out of all women who were registered pregnant that year
	1939–40	The charts of all pregnant women between 1939–40 in the District of Baltimore had their charts reviewed to see if they had a serological test for syphilis (1264 whites and 698 blacks). In 28.9% of whites and 6.8% of blacks no test was performed [115]. ASP is defined as the percent of those with a positive serological test out of all women who were registered pregnant that year
	1959–62	6 672 persons aged 18–79 sampled via a nationwide probability sample in the Health Examination Survey were tested for syphilis with the VDRL between 1959 and 1962. Comparisons by race are limited to NH white and NH black due to small sample sizes of other groups.[25]
	1976–1980	12 989 persons were tested for syphilis in National Health and Nutrition Examination Surveys-II—a stratified probability cluster survey of the US population conducted 1976–80. Testing was via the RPR with confirmation via the MHA-TP or the FTA [116]
	2001–04	5 767 participants (18- to 49-year-old) in the National Health and Nutrition Examination Surveys 2001–2004 were tested for syphilis IgG antibody using an EIA. Specimens with positive or indeterminate EIAs underwent RPR testing; RPR titers >1:8 were considered positive [26]
Zimbabwe	1945–49	Routine antenatal clients at Harare were tested for syphilis via the WR. Number tested not stated [117]
	1948–49	Routine antenatal clients at Umtali were tested for syphilis via the WR. Number tested not stated [117]
	1991	1433 pregnant women included in a study conducted in Umzingwane District. Syphilis testing with RPR test only [118]
	2002–03	Cross-sectional study of 691 pregnant women. Study was conducted from three peri-urban clinics around Harare. Syphilis testing with RPR and TPHA [119]
	2002–04	Cross-sectional study that enrolled pregnant women in Harare (n = 691) and Moshi (n = 2654). Syphilis testing with RPR and Determine Syphilis TP [120]
	2003	Pregnant women recruited for cross-sectional study in Gutu District of Zimbabwe. Syphilis testing with RPR test only [121]
	2005	Random sampling of 2969 pregnant women at Harare Maternity Hospital, Harare. Syphilis testing with RPR and TPHA [122]
	2009–11	WHO Global Health Observatory Data Repository

Abbreviations: EIA—Enzyme Immuno Assay, FTA—Fluorescent Treponemal Antibody test, MHA-TP—Microhemagglutination Assay for Treponema Pallidum, RPR—Rapid Plasma Reagin, TPI—Treponema Pallidum Immobilization Assay, TPHA—Treponema Pallidum Hemaglutination Assay, WR—Wasserman Reaction, VDRL—Venereal Diseases Research Laboratory, MHA-TP—Microhemagglutination Assay for Treponema Pallidum

The changes in ASP over time in each population were depicted graphically. A summary of the study characteristics used to provide these prevalence estimates is given in Table 1.

### National and regional variations in ASP in 1990–1999 and 2008

#### National ASP

1990–1999. National antenatal syphilis prevalence estimates for 1990 to 1999 were taken from an Institute for Health Metrics and Evaluation (IHME) database on the prevalence of syphilis in low risk populations compiled for the Global Burden of Diseases (GBD) study [28]. The IHME data sources included UNAIDS epidemiologic fact sheets, UNGASS country progress reports, reports from country specific surveillance systems, WHO reports on syphilis epidemiology, and data from correspondence with GBD collaborators. These were supplemented by a systematic literature review of syphilis seroprevalence (most recent PubMed search was October 2011). To be included, a study needed to provide data on the prevalence of syphilis in populations considered representative of the general population and have a sample size of at least 100. Data from high-risk populations (e.g. sex workers and STI clinic attendees) were excluded [6,28–32].

For each country we extracted the studies conducted in antenatal populations between 1990 and 1999 and used the median of these studies as a measure of the national prevalence of antenatal syphilis.

2008. ASP for 2008–2009 were taken from a review article on the prevalence on ASP conducted by the WHO and others [10]. In brief, data on syphilis seropositivity among ANC attendees for each country were taken from the WHO HIV Universal Access reporting system for 2008. If 2008 data were not available, then 2009 data was used. 97 of 193 countries had data available.

#### Regional ASP

Countries were grouped into 6 geographic regions, based on groupings used by the WHO [17], and the country median ASPs were used to compute the median ASP and interquartile range for each region for 1990–1999 and 2008.

### Case-reporting-based syphilis incidence (CSI)

We used various sources to obtain the number of cases of acquired syphilis per 100 000 per year for 8 of the 11 reviewed countries with available published data from surveillance data. Much of the SA and USA surveillance data was not disaggregated by race and thus we report both the overall national incidence and the more limited data available for the black and white racial groups in these countries. Details of the sources of data are provided in Table 2.

**Table 2:** Sources of data for incidence of syphilis as ascertained by number of cases reported to central authorities (all reported per 100,000 population per year).

Country	Sampling period	Reporting mechanism, stage of syphilis reported and study reference
Canada	1935	National incidence calculated by aggregating all provincial reports which are comprised of all cases of syphilis reported by private and state institutions [123]
	1944–1955	As of 1944 Canada commenced a national reporting system for all cases of syphilis. Figure provided is the annual reported rate of all acquired syphilis per 100 000 [124]
	1996 & 2003–2012	All cases of syphilis according to national reporting system [125]
Denmark	1915–51	All cases of acquired syphilis reported to national reporting system [126]
	1952–65	All cases of primary, secondary and early latent syphilis reported to national reporting system [3]
		All cases of acquired syphilis reported to national reporting system [127,128]
Japan	1947–56	Total number of acquired syphilis cases reported per annum to the Department of Health [94]
Norway	1915–51	All cases of acquired syphilis reported to national reporting system [126]
	2006–2009	All cases of acquired syphilis reported to national reporting system [127,128]
Singapore	1927–1955	All cases of primary, secondary syphilis reported to Ministry of Health by Social Hygiene Clinics [99]
	1974–84	All cases of primary, secondary and early latent syphilis reported to Ministry of Health [129]
South Africa	1939	All cases of acquired syphilis in the Cape Town magisterial district reported to the Medical Officer of Health for the year 1939. Incidence figures per 100 000 population are calculated separately for whites and blacks [72]
	1937–39	Cases of all stages of acquired syphilis reported to ten Magisterial Medical Officers of Health. Incidence per 100 000 were as follows: Johannesburg– 691, Cape Town– 851, Pretoria– 1645, Springs– 1474, Germiston– 4028, Benoni– 1167, Kimberly– 1854, Bloemfontein– 1004, Pietermaritzburg– 1337, Vereeniging– 1648; median value of 1474 used as syphilis incidence estimate for 1938 [130]
	1943	Cases of all stages of syphilis in workforce of Gold Mining companies [131]
	1946	Cases of all stages of syphilis in workforce of Gold Mining companies [131]
	1999	Estimated incidence of all cases of acquired syphilis for 1999 based on reported prevalence figures. Median prevalence rates were derived from published data generated by the Sexually Transmitted Infections Reference Centre (STIRC) and other research centres to cover as comprehensively as possible the whole country. Appropriate adjustments for differences in the prevalence of STIs were made for age (e.g. lower prevalence rates for 35–49 year age group), population groups and geographical regions (e.g. urban vs. rural) and gender (e.g. female infections more common) [132].
United Kingdom	1902–1950	Numbers of new diagnoses of syphilis (primary, secondary and early latent) reported in England, Wales and Scotland [133]
	1990–2012	All cases of acquired syphilis reported to national reporting system [127,128]
USA	1937–38	Rates of primary and secondary syphilis reported to CDC broken down into incidence figures for whites and blacks in 1981, 1990 and 1993. The figures for 1956 and 1969 are only broken down by white versus non white (PS syphilis per 100 000 in 1956: white– 1.6 and nonwhite 22.5; 1969: white– 3.3 and nonwhite– 54.4) [5]
	1941–2008	Data provided by CDC for total number of syphilis cases in all population groups (http://www.cdc.gov/std/stats08/tables/1.htm)

### Associations with ASP

#### Syphilis treatment efficacy

We used two proxy variables as measures of a country’s syphilis screening/treatment efficacy:

1. Health expenditure. The per capita total expenditure on health (purchasing power parity in US$) for 1990 and 2000 were used for the 1990–1999 and 2008 analyses respectively.
2. Antenatal clients screened/treated for syphilis. The percent of antenatal care attendees tested for syphilis at their first antenatal visit multiplied by the percent of those who tested positive who received appropriate therapy for syphilis. Where data was missing for percent receiving therapy we used regional medians. The data for these two variables was only available from 2006 until 2013. For each country, we used data from the first year data was available. The health expenditure and antenatal screening and therapy indicators were taken from the WHO Global Health Observatory Data Repository (http://apps.who.int/gho/data/node.main.A1358STI?lang=en).

#### GDP per capita

To control for economic development we use Gross Domestic Product (GDP) per capita (US$ Purchasing Power Parity). We use the GDP from 1995 for the 1990–1999 analysis and 2005 for 2008 analyses. The data for these variables are taken from the Human Development Reports [33,34].

#### Circumcision

The national prevalence rates of male circumcision as of December 2006 were taken from a World Health Organization and Joint United Nations Programme on HIV/AIDS publication. These estimates were based on Demographic and Health Survey data or from other published sources [35]. Countries were classified as having circumcision prevalence rates <20%, 20–80% or >80%.

#### World regions

WHO world regional variables were included to test for regional differences in ASP [6].

### Statistical analysis

The estimated prevalence of active syphilis can vary according to whether it is estimated via a treponemal or a nontreponemal test or a combination of the two [36]. To account for this, we applied recently published correction factors to adjust the ASP estimates for the 13 population longitudinal analysis and for the 1990–99 estimates according to the testing algorithm used [36]. These correction factors are based on a systematic review and meta analysis that estimated the proportion of pregnancies with “probable active syphilis” based on different kinds of tests from studies that conducted both treponemal and nontreponemal testing on samples [36]. Studies that use both a treponemal and a nontreponemal test to diagnose infection were regarded as offering the most accurate measure of active syphilis prevalence and no correction factor was applied. Studies using only a treponemal test will falsely label persons with old or treated syphilis as having active syphilis and a correction factor of 0.536 was applied to these prevalence estimates. Studies using only nontreponemal testing will falsely diagnose active syphilis in persons with other inflammatory conditions that lead to biological false positive reactions in the nontreponemal test. A correction factor of 0.522 was applied to these prevalence estimates. In addition the study recommended the use of a correction factor of 0.686 for studies that did not report the type of testing used. This was calculated as the average of the two correction factors noted above (0.536, 0.522). The 2008 ASP estimates included a correction factor that similarly estimated the prevalence of active syphilis. All ASPs reported and used in analyses therefore refer to the adjusted estimates. Simple and multiple linear regression was used to evaluate the relationship between syphilis prevalence and each of the explanatory variables. The analyses were conducted separately with syphilis prevalence for 1990–1999 and 2008 as the outcome variables. A brief historical overview of the serological diagnostic tests used to assess syphilis prevalence and their diagnostic accuracy is provided in Box 1. All analyses were performed in STATA 13.0 (StataCorp LP, College Station, TX, USA).

**Box 1. A brief historical overview of the serological diagnostic tests used to assess syphilis and their diagnostic accuracy**

**Diagnostic tests**

Serologic testing is the most important modality used to make the diagnosis of syphilis. The serological tests for syphilis come in two categories:

- Nontreponemal tests (NTT) that do not directly detect antibodies to *T*.*pallidum* but rather reactions to the inflammation that *T*.*pallidum* infection produces. In general these tests reflect the activity of the infection and are used to monitor response to therapy. Examples of nontreponemal tests include: Wasserman reaction (WR), Kahn Test (KT), Venereal Disease Research Laboratory (VDRL), Rapid Plasma Reagin (RPR) and the Toluidine Red Unheated Serum Test (TRUST).
- Specific treponemal tests (TT) detect antibodies to specific *T*.*pallidum* antigens or collections of antigens. Examples of these tests include: Microhemagglutination test for antibodies to *T*. *pallidum* (MHA-TP), Fluorescent treponemal antibody absorption (FTA-ABS), *T*. *pallidum* particle agglutination assay (TP-PA) and the *T*.*pallidum* enzyme immunoassay (TP-EIA).

A complement-fixation-based test, the Wasserman reaction was the first serological test used in the diagnosis of syphilis [77]. The antigens used in the test were originally derived from tissue extracts of livers from infants who died of presumed syphilis. Subsequently a bovine heart extract was used and to this day remains the basis of the source of the cardiolipin used in this test [78]. A significant problem with the early NTTs was the high rate of false positives they produced [79]. In order to improve the specificity of these tests cholesterol and lecithin were added [78]. The complement-fixation-based tests were difficult to perform and time consuming and thus Kahn’s development of a flocculation test without complement that could be completed within a few hours represented a major advance [80]. The resultant Kahn Test (KT) was modified in a number of subsequent NTTs but all these tests suffered from the lack of a standardized antigen [80]. It was only in 1941 that a way was found to precipitate cardiolipin from bovine hearts in a manner that made standardization possible [80]. This in turn paved the way for the development of microflocculation tests such as the VDRL in 1946 which yielded more reproducible results than preceding tests [81]. The RPR, which was developed in the 1950s, incorporated charcoal microparticles into the reagent to facilitate reading the test [78]. The toluidine red unheated serum test (TRUST) and other NTTs represented further modifications of the RPR. Most recently a range of automated enzyme immunoassays and chemiluminescene assays have been developed with good diagnostic accuracy (Table 3) [82].

**Testing algorithms**

In the traditional testing algorithm, the NTT are used as the initial test to screen for syphilis and the TT are used to confirm positive cases. In 2008 it was first reported that a number of laboratories had reversed this testing order and were screening with TT and confirming infection with NTT [83]. This reversed testing algorithm approach has been implemented in a number of settings [82].

**Table 3:** Sensitivity and specificity of various treponemal and nontreponemal tests. Figures in parentheses refer to the range of sensitivities and specificities found in the included studies (Table is based on data from [78] and [82]).

	Sensitivity at various syphilis disease stages (%)				Specificity (%)
	Primary	Secondary	Early Latent	Late Latent	
Nontreponemal Tests					
VDRL	78(74–87)	100	95(88–100)	71(37–94)	98(96–99)
RPR	86(77–100)	100	98(95–100)	73	98(93–99)
USR	80(72–88)	100	95(88–100)		99
TRUST	85(77–86)	100	98(95–100)		99(98–99)
Early Treponemal Tests					
FTA-ABS	84(70–100)	100	100	96	97(94–100)
MHA-TP	76(69–90)	100	97(97–100)	94	99(98–100)
TPPA	88(86–100)	100	100	NA	96(95–100)
TPHA	86	100	100	99	96
Enzyme immunoassays					
IgG ELISA	100	100	100	NA	100
IgM-EIA	93	85	64	NA	NA
ICE	77	100	100	100	99
Immunochemiluminescence assays					
CLIA	98	100	100	100	99

Abbreviations: CLIA, chemiluminescence assay; ELISA, enzyme-linked immunosorbent assay; EIA, enzyme immunoassay; FTA-ABS, fluorescent treponemal antibody absorption assay; ICE, immune-capture EIA; MHA-TP, microhemagglutination assay for Treponema pallidum; NA, not available; TPHA, T. pallidum hemagglutination assay; TPPA, T. pallidum particle agglutination; TRUST,toluidine red unheated serum test, USR, unheated serum regain; VDRL, Venereal Disease Research Laboratory.

## Results

### Longitudinal changes in ASP in 13 populations

**Fig 2:**
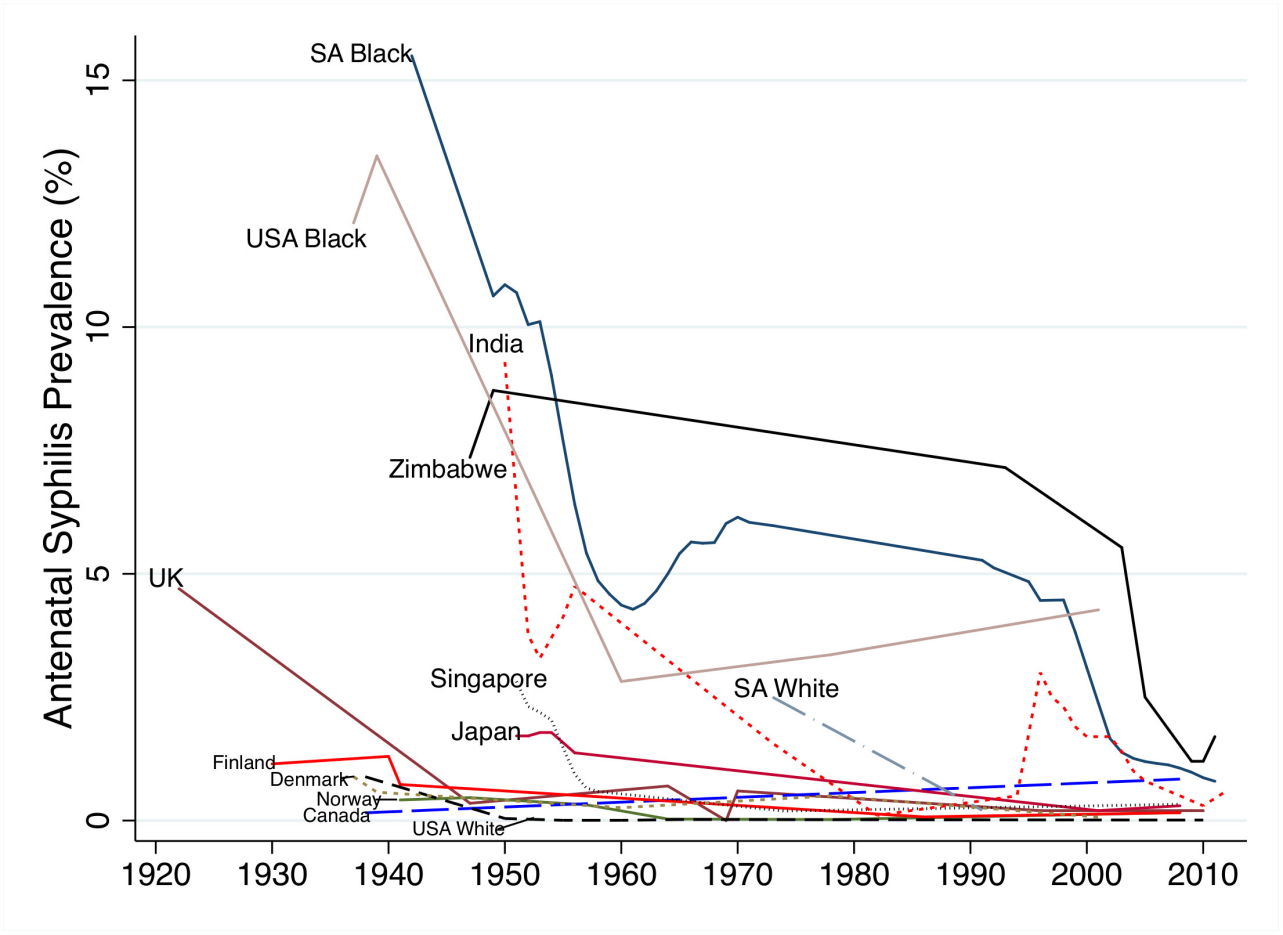
Changes in Antenatal Syphilis Prevalence in 13 populations between 1922–2012.

ASP declined in all populations assessed in this period (Fig 2) but at different times and rates. In the case of Canada, Denmark, Finland, Norway and whites in the USA, ASP dropped to below 1% before 1945 when penicillin started to become widely available. We found no data points for the UK between 1922 and 1947 but by 1947 its ASP had declined to 0.4%. By 1955 ASP in Singapore and Japan had declined to below 2%. There was little data available for whites in SA but in a small sample in 1972, prevalence was 2.5% and at the time of the first large sample (1991) ASP was under 1%. ASP declined somewhat more slowly in India (1.6% in 1973 and 0.1% in 1982), and USA blacks (3.4% in 1978 and 4.2% in 2001). In Zimbabwe and black South Africans, ASP was high in the pre penicillin period, dropped in the post-penicillin period but then plateaued at around 6% until the end of the 20th century when ASP dropped precipitously to just above 1%.

### Syphilis case incidence (CSI)

**Fig 3:**
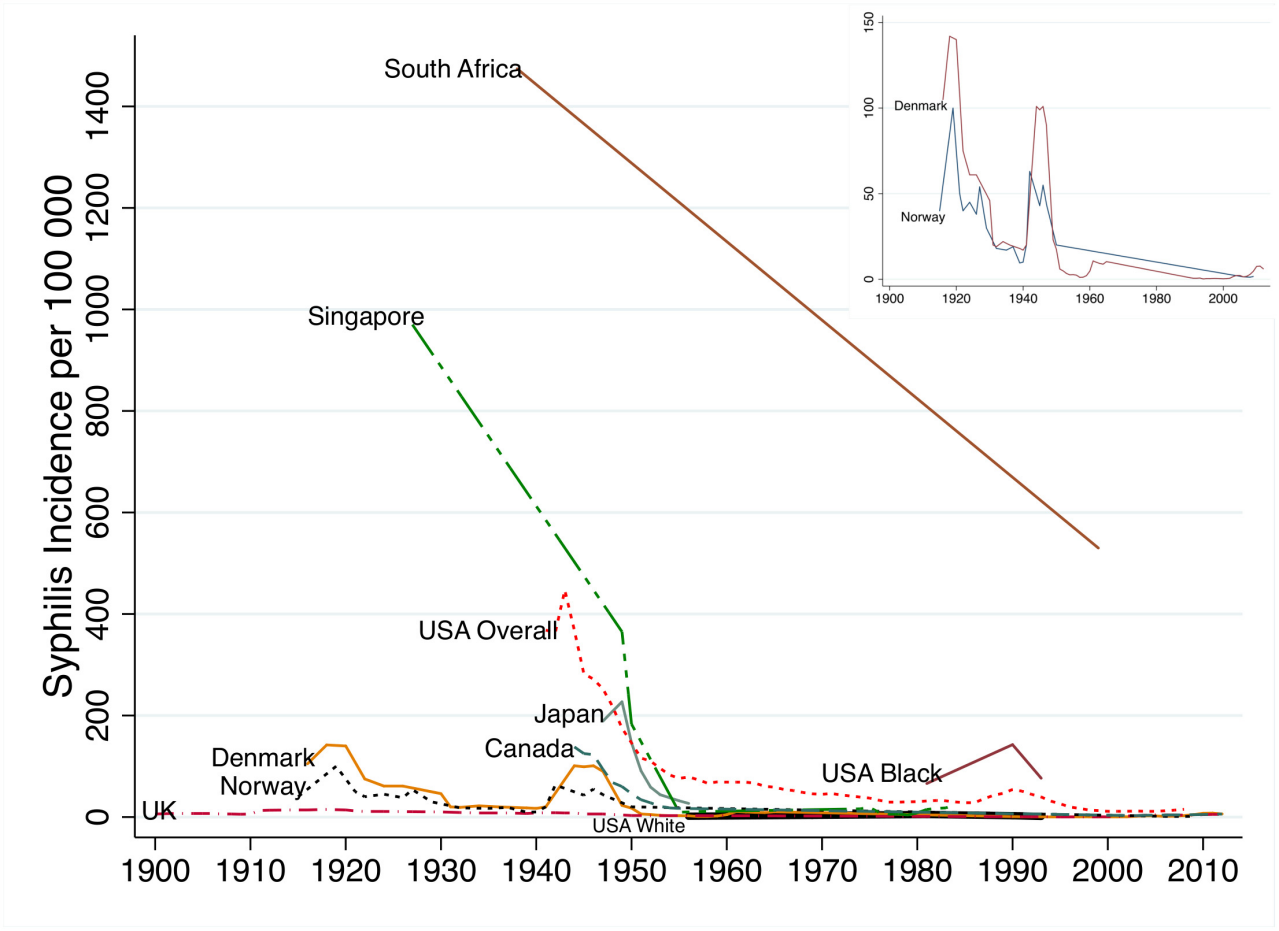
Changes in case based reported syphilis incidence (cases per 100 000) in nine populations between 1900–2012 (Inset syphilis incidence in Denmark and Norway).

Changes in incidence of reported cases of syphilis per year per 100 000 population were found to be broadly commensurate with ASP figures (Fig 3 and Table 2). Denmark, Norway and the UK had low syphilis incidence prior to the introduction of penicillin. By 1939 all three countries had an incidence below 20. After brief increases during the Second World War, incidence in these countries as well as Canada, Japan, Singapore and the USA (overall population) declined rapidly in the post-war period. By 1956 the incidence in Denmark, Norway, Canada, Japan, Singapore, UK and whites in the USA was below 30/100 000. Incidence in blacks in the USA remained approximately an order of magnitude greater than whites at all time points for which we have data. Incidence in black South Africans in 1939 was five times higher than whites. The median incidence in ten cities in South Africa (overall population) between 1937 and 1939 was 1474 (range 691–4028). This declined somewhat to an estimated 530 by 1999 and more precipitously after 1999 in keeping with the contemporaneous declines in ASP [15,37]. Numbers of cases diagnosed with syphilis in Zimbabwe were high in all reports in the 20^th^ century [38–41] but we have not reported incidence as population denominators were not provided and a number of expert reviews found evidence of both extensive misclassification of chancroid as syphilis [20,38,40] and endemic treponemes causing false positive serological diagnoses of syphilis [40].

### Risk factors associated with ASP

In both 1990–99 and 2008 on bivariate analyses, ASP was negatively associated with GDP per capita, health expenditure and residence in sub- Saharan Africa (henceforth termed Africa; Table 4). On multivariate analysis in both time periods, ASP was only associated with residence in Africa (Table 5). We found no evidence of an association between GDP per capita, health expenditure, screening/treatment or circumcision prevalence and ASP.

**Table 4:** Median (interquartile range) adjusted antenatal syphilis prevalence for the six WHO world regions. Statistical comparisons are Kruskal-Wallis tests comparing adjusted median ASP in each region with that in sub-Saharan Africa.

Region	n	1990–1999	n	2000–2009
Sub-Saharan Africa	33	3.01 (1.39–4.42)	35	1.48 (0.72–2.60)
Europe	14	0.09 (0.09–0.13)***	11	0.08 (0.04–0.70)***
East Mediterranean	10	0.38 (0.16–0.95)***	7	0.04 (0.00–0.71)**
Americas	12	0.59 (0.09–2.48)*	21	0.54 (0.35–0.93)*
South/South East Asia	6	1.25 (0.66–1.68)	7	0.40 (0.02–1.47)
Western Pacific	12	0.42 (0.20–1.35)**	9	0.21 (0.10–1.53)

*P<0.05,

** *P*<0.005,

*** *P*<0.0005

**Table 5:** Bivariate and multivariate regression analyses of the relationship between adjusted national antenatal syphilis prevalence and putative risk factors including regional dummies. Beta-coefficients (95% Confidence Intervals).

	1990–1999			2008–2009		
	n	Bivariate	Multivariate	n	Bivariate	Multivariate
GDP (1995/2005)	80	-0.0001 (-0.002- -5.93e-0.6)*	0.003 (-0.00006–0.0006)	87	-0.00006 (-0.0001–0.00002)**	-0.00005 (-0.0002–0.00007)
Health Expenditure 1990/2000	86	-0.008 (-0.002- -3.03e-06)*	-0.005 (-0.01–0.001)	88	-0.0008 (-0.002- -0.0001)*	-0.001 (-0.01–0.36)
Screening/treatment efficacy		NE	NE	82	-0.007 (-0.02–0.0006)	-0.09 (-0.52–0.35)
Circumcision	85	0.04 (-0.39–0.48)	-0.28 (-0.02–0.008)	85	0.15 (-0.15–0.44)	0.0004 (-0.001–0.002)
Europe	87	R	R	90	R	R
Africa		2.7 (1.7–3.7)**	2.9 (1.2–4.7)**		1.46 (0.59–2.34)**	1.45 (0.31–2.59)*
Eastern Mediterranean		0.2 (-1.0–1.5)	0.6 (-2.0–3.2)		0.12 (-1.10–1.35)	0.09 (-1.38–1.57)
Americas		1.2 (-0.4–2.4)	1.3 (-0.5–3.2)		0.54 (-0.40–1.49)	0.25 (-0.91–1.43)
South East Asia		1.4 (-0.7–3.7)	1.6 (-0.5–3.7)		0.63 (-0.59–1.86)	0.36 (-1.11–1.80)
Western Pacific		0.5 (-0.7–1.7)	0.6 (-1.4–2.7)		0.76 (-0.38–1.90)	0.45 (-1.01–1.90)
N			58			73

*P < 0.05,

** P < 0.05

NE—Not entered

n refers to number of observations in the bivariate analyses

**Fig 4:**
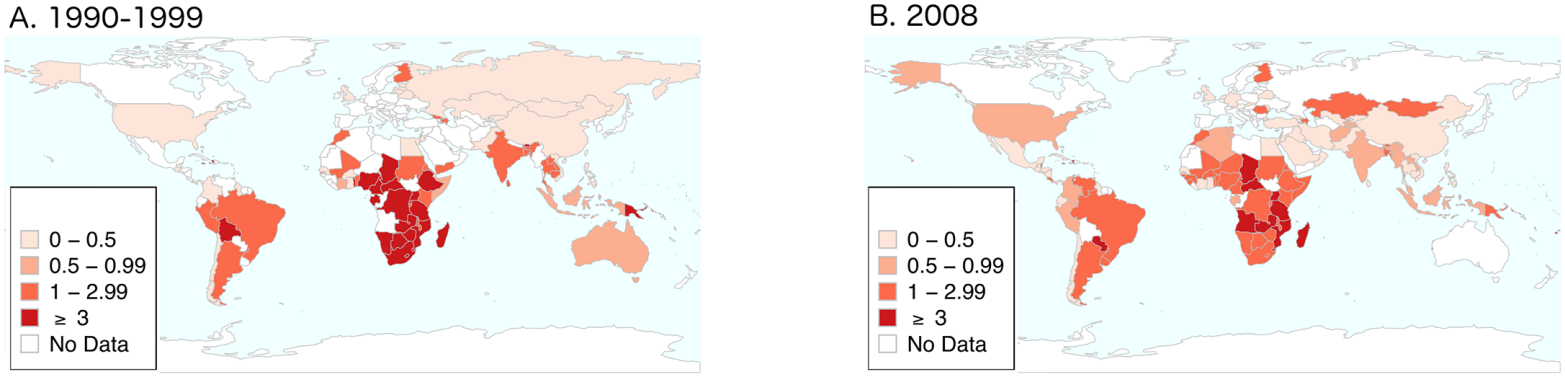
Antenatal syphilis prevalence by country in 1990–99 (A) and 2008 (B).

Differences in national ASP in the two time periods are represented graphically in Fig 4.

### Variations in regional ASP

By 1990–99, ASP had dropped to a median 3.0 (IQR 1.4–4.4) in Africa versus 0.1 (IQR 0.1–0.1) in Europe, 0.4 (IQR 0.2–1.0) in the East Mediterranean, 0.4 (IQR 0.2–1.4), 0.6 (IQR 0.1–2.5) in the Americas, 1.3 (IQR 0.7–1.7) in South/South Eastern Asia and in the Western Pacific. ASP was statistically significantly higher in Africa in 1990–1999 than in the other world regions except South/South East Asia where this relationship was not statistically significant (Table 4). By the following decade the median prevalence of ASP had fallen in most regions including Africa but Africa’s median ASP remain statistically significantly higher than the ASP in Europe, Eastern Mediterranean and the Americas.

Sensitivity analyses were conducted using the uncorrected ASPs. This did not alter the bivariate and multivariate results.

## Discussion

What determines the tremendous variation in syphilis prevalence around the world over the past century? Our study provides no definitive answers but contributes some clues. Our 11 country longitudinal case study found that although syphilis prevalence declined in all populations investigated in the post-penicillin period, it did so to varying degrees and at different times. In Canada, Denmark, Finland, Norway, UK and USA whites, ASP declined to low levels prior to the introduction of penicillin. It could be argued that this was due in part to earlier and better access to efficacious therapies such as salvarsan which had been available since 1909 [42,43]. It is however striking how much lower ASP was in these populations in the pre- penicillin period than they were in South Africa and Zimbabwe 50 years into the penicillin period. The data from CSI shows a similar pattern. When plotted by themselves the case-based-reporting epidemic curves of countries like Denmark and Norway reveal dramatic changes in syphilis incidence coinciding with the two world wars and the introduction of penicillin (Fig 3, inset [3]). However, when plotted on the same axes as the high ASP populations these fluctuations are dwarfed by the large difference in prevalence between high and low prevalence populations. We have only data available for 11 countries and thus should be careful about the conclusions we draw from this small dataset. The data is however broadly commensurate with other sources. For example, although our case study contained only two countries from Africa, a review of longitudinal changes in ASP from 11 Southern and Eastern African countries from the pre-AIDS period found ASP in the same range (median ASP 10%; Interquartile range, 6.5–11.5% [44]) as those found for Zimbabwe and black South Africans from the same period reported in the current study. Our findings are also commensurate with those from a review conducted by the WHO for the years 1945 to 1958, which found that the incidence of syphilis declined in all regions of the world excluding Africa [21]. This review was based on reported cases of syphilis per year. As noted above, this indicator is a far from optimal marker of syphilis incidence. Using ASP data, our review of the global epidemiology of syphilis comes to a similar conclusion: the decline in ASP was slowest in Africa. Other non-African low and middle-income countries (LMICs) such as India and Singapore (which was a middle income country in the 1950s [45]) experienced a more rapid decline in ASP. The syphilis prevalence in both 1990–99 and 2008 was significantly higher in Africa than most other regions. This finding remained after controlling for GDP, health expenditure, syphilis testing/treating and circumcision.

The reason why ASP was not uniformly raised in LMICs but predominantly in Africa requires further research. The fact that the global distribution of syphilis is similar to that of HIV and Herpes Simplex Virus-2 (HSV-2) may be instructive [32,46–48]. All three STIs have been found to be more prevalent in Africa than elsewhere [48,49]. In studies that used the same national ASP data in 1990–99 as those used here, ASP was found to be closely correlated with both HSV-2 prevalence in 1990–99 [47] and subsequent peak HIV prevalence [32]. Whilst the association of HIV and syphilis could conceivably be due to syphilis enhancing the transmission of HIV this cannot easily explain the association between HSV-2 and syphilis—HSV-2 and syphilis have not been shown to influence the prevalence of one another. The association between these two is thus likely due to a common factor driving both STIs. The fact that HSV-2 prevalence should not be influenced by STI treatment capacity makes it less likely that this common factor is STI treatment capacity.

Other types of evidence suggest that behavioural factors that influence sexual network structure may constitute an important common risk factor for HIV, HSV-2 and syphilis. A number of studies have found evidence that more connected sexual networks play a role in generating higher prevalences of syphilis, HIV, HSV-2 and other STIs [50–52]. There is some evidence that the prevalence of syphilis and the other STIs are correlated with the markers of network connectivity such as the prevalence of sexual partner concurrency [50,51,53–55]. One hypothesis that could tie together these results is therefore that populations with more connected sexual networks had, as a result, higher prevalences of HSV-2 and syphilis in the pre-AIDS period. These populations then went on to have higher prevalences of HIV explaining the positive association between pre-AIDS syphilis/HSV-2 prevalence and peak HIV prevalence. There are however several important studies that have contested the association between STIs and markers of network connectivity and thus further studies are required to confirm or refute this hypothesis [56–58]. Other factors that may have played a role in the differential decline of ASP include differential access to appropriate health care [59], usage of barrier contraception [60], the presence or absence of social disruption and other socio-economic factors such as the extent of social capital and the degree of economic inequality [59,61,62].

There are numerous limitations of this study, including the poor quality of much the data used in this study. There is a paucity of population-based samples of ASP from prior to the 1990’s. Our linear regression analyses are compromised by the low number of data points for both ASP and the independent variables that were available from the 1980s and 1990s. Our measures of syphilis treatment efficacy are far from optimal but were the best we could find sufficient data for. In addition, they are similar to WHO estimates for the proportion of all persons with symptomatic primary and secondary syphilis in 1995 who received appropriate therapy as determined by WHO estimates [8]. According to these estimates this proportion varied between 0.35 in Africa and South/South East Asia, 0.6 in East Asia, Latin America, East Europe, the Middle East and 0.85 in North America, Western Europe and Australasia. They are also close to 2008 estimates for the proportion of all ANC attendees who are tested and treated for syphilis according to the study by Cal Ham *et al* [36] (Africa and Eastern Mediterranean, 0.3; South/South East Asia, 0.4; Americas and Western Pacific, 0.6 and Europe 0.7). We were unable to find sufficient comparable data on aspects of sexual behavioural and condom usage data to be able to include these in our explanatory models. The use of ASP as a measure of syphilis prevalence has three major limitations. Firstly, there is a paucity of published large surveys of ASP particularly from the early period of this study [63]. Secondly, there are a number of different tests and different testing algorithms for the diagnosis of syphilis, each of which is susceptible to differing rates of false positive and negative results. In countries where conditions such as the endemic trepanematoses are endemic a large proportion of positive results may then be false positives [64] since these give positive syphilis test results. Thirdly, antenatal populations differ systematically from general populations in a number of ways [36,65]. An example is that syphilis has been shown to impair fertility and thus ASPs may underestimate the prevalence of syphilis in the general population [66]. Unlike syphilis, HIV prevalence estimates from population surveys have been compared with those obtained from antenatal surveys. In two countries where the HIV epidemic had stabilized (Uganda and Zimbabwe) the estimates were found to be very similar [65,67] whereas in four other countries (Ethiopia, Kenya, Malawi, Tanzania) the population estimates were lower than those from the antenatal surveys [68]. We use two strategies to overcome the first two limitations.

Firstly, for the cross-country quantitative comparative analyses we limit the analyses to the periods 1990–1999 and 2008. This has three advantages. (1) By the 1990s the endemic treponemes had been eradicated from most of the world’s populations [69,70]. (2) The syphilis tests and testing algorithms used in this period resulted in fewer false positive results than those used in earlier periods [36]. (3) There is also better ASP data available for this time period than earlier periods [10]. Secondly, we conduct sensitivity analyses that correct for the type of syphilis testing algorithm used.

The ASP estimates for our 11-country analysis were derived using different methodologies which limits the validity of comparing estimates within and between countries. The nontreponemal tests used from early on in the study period were particularly susceptible to false positive results which could falsely inflate ASP estimates from this period [71]. In addition, the sample size of 11 countries is too small a sample to draw firm conclusions from. The ASP estimates from our 11-countries are however broadly commensurate with other published data. The incidence of syphilis (as assessed by cases per year) has also been shown in a number of studies to be approximately an order of magnitude higher in black as compared to white Americans and South Africans in data from the pre penicillin period up to contemporary times [5,72–75]. Finally, we do not include uncertainty estimates of our ASP or CSI data and we do not control for differences in age structures of the sampled populations.

### Conclusion

By 1959, 600 tons of penicillin were being produced per year and there is little doubt that its widespread use played an important role in the decline of syphilis rates [21]. However, ASP in certain populations around the world exhibited more resilience in response to the introduction of penicillin and associated syphilis control strategies. The populations that in the 1990’s had high prevalences of syphilis and HSV-2 went on to have high HIV prevalences [52]. Although this analysis suffers from numerous limitations, if taken in conjunction with the other evidence reviewed here it generates the hypothesis that more connected sexual networks may have been partly responsible for the higher prevalences of syphilis and other STIs in high prevalence populations. High AIDS related mortality has been shown in both MSM in the USA and the generalized HIV epidemics of Africa to have disrupted sexual networks and thereby reduced the prevalence of syphilis [12–14]. It is possible that the widespread use of antiretroviral therapy may be followed by the reconstitution of more connected sexual networks and a return of syphilis in these populations—as has occurred in MSM in the USA and elsewhere [76]. An improved understanding of the factors underpinning variations in syphilis rates around the world in the last 100 years could help us to better understand the current and predict the future patterning of syphilis prevalence.

## Supporting Information

S1 Table PRISMA checklist. (DOC)
